# Fabrication and Investigation of Acid Functionalized CNT Blended Nanocomposite Hollow Fiber Membrane for High Filtration and Antifouling Performance in Ultrafiltration Process

**DOI:** 10.3390/membranes13010070

**Published:** 2023-01-05

**Authors:** Eunmok Yang, Shinyun Park, Yeji Kim, Numan Yanar, Heechul Choi

**Affiliations:** 1 School of Earth Sciences and Environmental Engineering, Gwangju Institute of Science and Technology (GIST), 261 Cheomdangwagi-ro, Buk-gu, Gwangju 61005, Republic of Korea; 2 Department of Civil and Environmental Engineering, Colorado State University, Fort Collins, CO 80523, USA; 3 Department of Civil, Architectural, and Environmental Engineering, University of Texas at Austin, Austin, TX 78712, USA

**Keywords:** nanocomposite, hollow fiber membrane, carbon nanotubes, functionalization, ultrafiltration, antifouling

## Abstract

In this study, we fabricated a nanocomposite polyethersulfone (PES) HF membrane by blending acid functionalized carbon nanotubes (FCNT) to address the issue of reduced membrane life, increased energy consumption, and operating costs due to low permeability and membrane fouling in the ultrafiltration process. Additionally, we investigated the effect of FCNT blending on the membrane in terms of the physicochemical properties of the membrane and the filtration and antifouling performance. The FCNT/PES nanocomposite HF membrane exhibited increased water permeance from 110.1 to 194.3 LMH/bar without sacrificing rejection performance and increased the flux recovery ratio from 89.0 to 95.4%, compared to a pristine PES HF membrane. This study successfully developed a high filtration and antifouling polymer-based HF membrane by blending FCNT. Furthermore, it was validated that blending FCNT into the membrane enhances the filtration and antifouling performance in the ultrafiltration process.

## 1. Introduction

Membrane-based water treatment has attracted considerable attention because of its high cost-effectiveness, low energy consumption, low carbon footprint, and easy coupling with other water treatment technologies [1,2,3,4]. Among membrane-based water treatment, including microfiltration (MF), ultrafiltration (UF), nanofiltration (NF), and reverse osmosis (RO), UF has been greatly studied owing to its wide utilization in the treatment of drinking water and wastewater, hemodialysis, protein harvesting, and water pretreatment for desalination plants [5,6].

The hollow fiber (HF) membrane configuration has distinct advantages, including a high surface area per unit module volume, self-mechanical support, and easy scale-up, compared with other membrane configurations [7]. Owing to their special characteristics, HF membrane configurations are widely used in various membrane-based separation processes, including water treatment, desalination, hemodialysis, pervaporation, gas separation, and organic solvent nanofiltration [8]. The HF membrane configuration is mainly used in the UF process [9,10].

Polymer-based membranes such as polyethersulfone (PES), polysulfone (PS), polyvinyl difluoride (PVDF), and polyacrylonitrile (PAN) are mainly used in the UF process. In particular, PES has been highlighted as a membrane material owing to its high mechanical strength, excellent thermal stability, remarkable chemical resistance, easy processability, and low cost [1,6,9]. However, despite the many advantages mentioned above, low permeance and membrane fouling due to the hydrophobic nature of PES causes a reduced membrane lifespan, increasing energy consumption and creating higher operating costs [1,11]. Therefore, various methods for increasing hydrophilicity have been studied to overcome the low permeance and membrane fouling problems, including hydrophilic material coating, grafting, and blending [12,13,14]. Among these, the blending of hydrophilic nanomaterials with polymers to prepare nanocomposite membranes has been actively researched by academia and industry owing to its feasible processability and easy scale-up [15,16]. For example, carbon-based nanomaterials such as carbon nanotubes [17,18], graphene oxide [19,20], and carbon quantum dots [21,22] have been widely used to improve the permeance and antifouling properties of membranes. Metal [23,24] and metal oxide [25,26] blended PES membranes also exhibited improved filtration performance and antimicrobial properties. In addition, it was recently found that porous nanomaterials such as zeolite [27,28], metal–organic frameworks [29,30], and mesoporous carbon [31] increase the filtration performance of PES membranes.

Considering all the aspects mentioned above, nanocomposite PES HF membranes are expected to have high potential for the UF process. Therefore, in this study, a nanocomposite PES HF membrane was fabricated by blending acid functionalized carbon nanotubes (FCNT) to improve the filtration and antifouling performance in the UF process. FCNT were selected as blending nanomaterial owing to their unique physicochemical properties such as hydrophilicity, negative surface charge, and excellent chemical and thermal stability [17,18,32]. We systematically investigated the effect of FCNT blending on the physical and chemical properties of PES HF membranes. Subsequently, the filtration and antifouling performance of the fabricated PES HF membrane were evaluated to confirm the effect of FCNT blending on membrane performance. It was found that the FCNT/PES nanocomposite HF membrane exhibited higher filtration and antifouling performance than a pristine PES HF membrane in the UF process. Therefore, we successfully fabricated a high permeance and antifouling FCNT/PES nanocomposite HF membrane and validated that FCNT blending improved the filtration and antifouling performance of the PES HF membrane in the UF process.

## 2. Materials and Methods

### 2.1. Materials

Nitric acid (ACS reagent, 70%) and sulfuric acid (ACS reagent, 95.0–98.0%) were purchased from Sigma-Aldrich (Saint Louis, MO, USA). The raw multi-wall carbon nanotubes (abbreviated as CNT, CM-95, 93–97%) were purchased from Hanwha-nanotech (Seoul, Republic of Korea). PES (Veradel^®^ 3000 P, Brussels, Belgium) with a molecular weight 62,000–64,000 g/mol, was obtained from Solvay Specialty Polymers. N-methyl-2-pyrrolidinone (NMP, anhydrous 99.5%) and polyvinylpyrrolidone (PVP) with an average molecular weight 10,000 g/mol, were purchased from Sigma-Aldrich. Humic acid sodium salt (HA, technical grade) was purchased from Sigma-Aldrich for filtration and antifouling performance tests.

### 2.2. Acid Functionalization of CNT

**Figure 1:**
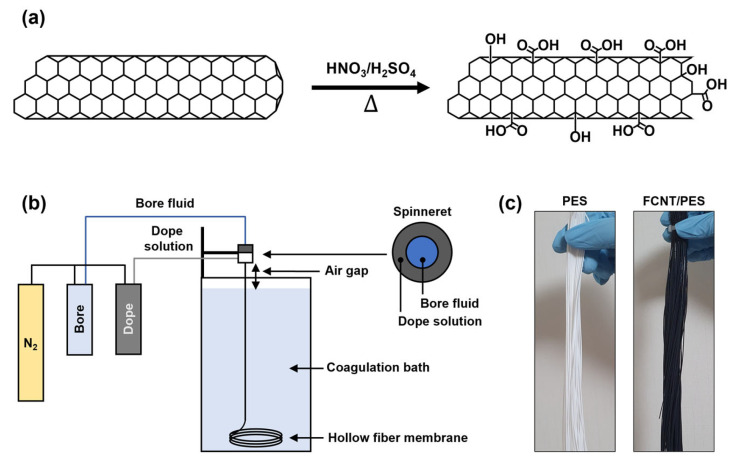
Schematic illustrations of FCNT/PES nanocomposite HF membrane preparation. (**a**) Acid functionalization of CNT. (**b**) Lab-scale dry-jet wet spinning process. (**c**) Photographic images of PES and FCNT/PES nanocomposite HF membranes.

The CNT were functionalized by the chemical oxidation method described in our previous report (Figure 1a) [17,32]. First, raw CNT were vigorously stirred in a 3:1 (*v*/*v* %) mixture of nitric acid and sulfuric acid under reflux at 100 °C for 3 h to remove any impurities. Afterward, the mixture with CNT was neutralized with DI water and a 0.45 μm nylon filter was used to filter the CNT. Subsequently, the filtered CNT were dried overnight in an oven at 100 °C. After drying, the CNT were immersed in the same acid mixture, followed by ultrasonication at 90 °C for 3 h to attach carboxylic acid functional groups onto the CNT. After the reaction, the functionalized CNT were neutralized and filtered. Finally, the filtered CNT were dried overnight in an oven at 100 °C to obtain FCNT.

### 2.3. Preparation of FCNT/PES Nanocomposite Hollow Fiber (HF) Membranes

To prepare the dope solution, the desired amount of PVP was dissolved in NMP. Subsequently, a predetermined amount of FCNT was dispersed in the PVP/NMP solution by ultrasonication at 50 °C for 90 min. PES was then added, and the mixture was vigorously stirred at 50 °C for 48 h. The composition of the dope solutions for the preparation of the FCNT/PES nanocomposite HF membranes is listed in Table 1. The dope solutions were degassed overnight in an oven at 50 °C and cooled to room temperature before spinning.

FCNT/PES nanocomposite HF membranes were prepared via a lab-scale dry-jet wet spinning process (Figure 1b). The bore fluid and dope solution were fed into the inner and outer channels of a single-layer spinneret, respectively. The two fed fluids met at the tip of the spinneret. Subsequently, they passed through an air-gap region and entered the coagulation bath. The spinning parameters for the FCNT/PES nanocomposite HF membranes are presented in Table 2.

After spinning, the as-spun HF membranes were soaked in DI water for 48 h to remove residual reagents. To prevent membrane shrinkage and pore collapse, the membranes were post-treated with a 50 wt% glycerol aqueous solution for 48 h and dried under ambient conditions (Figure 1c). Finally, the post-treated HF membranes were mounted onto a module with epoxy potting sealing to evaluate filtration performance.

### 2.4. Characterization

The microstructure of the CNT, FCNT, and HF membranes was observed using field-emission scanning electron microscopy (FESEM; Gemini 500, Zeiss, Oberkochen, Germany). Attenuated total reflection–Fourier transform infrared spectroscopy (ATR-FTIR; Hyperion 2000, Bruker, Billerica, MA, USA) was used to confirm the functional groups of the CNT, FCNT, and HF membranes. The surface chemical composition of the CNT, FCNT, and HF membranes was measured using X-ray photoelectron spectroscopy (XPS; NEXSA, Thermo Fisher Scientific, Waltham, MA, USA) with monochromatic Al Kα radiation.

The pore size and porosity of the inner surface of the HF membranes were investigated by surface FESEM image analysis using ImageJ 1.53k software [33,34]. The overall porosity (ε) of the HF membranes was determined by the dry–wet method and calculated using the following equation:

(1) $$\varepsilon =\frac{\frac{\left(m_{w}-m_{d}\right)}{\rho_{w}}}{\frac{\left(m_{w}-m_{d}\right)}{\rho_{w}}+\frac{m_{d}}{\rho_{m}}},$$

where $m_{w}$ and $m_{d}$ are the weights of the wet and dry membranes, respectively, $\rho_{w}$ is the density of DI water (1.00 g/cm^3^), and $\rho_{m}$ is the density of the PES polymer (1.37 g/cm^3^).

The hydrophilicity of the HF membranes was investigated by measuring their water contact angles using a contact angle analyzer (Phoenix 300, SEO Company, Suwon, Republic of Korea). The surface roughness of the inner surface of the HF membranes was measured in the tapping mode using an atomic force microscope (AFM, XE-100, Park systems, Suwon, Republic of Korea).

### 2.5. Filtration and Antifouling Performance

The filtration performance of the fabricated HF membranes was tested using a lab-scale cross-flow filtration system at room temperature (23 ± 2 °C). The all-filtration performance was measured after membrane stabilization at 4 bar for 30 min. The water flux (J, Lm^−1^ h^−1^ or LMH) and permeance (P, Lm^−1^ h^−1^ bar^−1^ or LMH/bar) of each HF membrane module were determined using the following equations:

(2) $$J=\frac{V}{A_{m}\Delta t},$$

(3) $$P=\frac{J}{\Delta P},$$

where *V* (L) is the permeated volume, *A_m_* (m^2^) is the effective membrane area of the HF membrane module, Δt (h) is the filtration time, and ΔP (bar) is the transmembrane pressure.

The rejection and antifouling performance were evaluated using an HA solution. The rejection (R, %) of each HF membrane module was calculated using the following equations:

(4) $$R=\left(\frac{C_{f}-C_{p}}{C_{f}}\right)\times 100\%,$$

where *C_f_* and *C_p_* are the HA concentrations of the feed and permeate solutions, respectively. HA concentration was determined using a UV-Vis spectrophotometer (Optizen POP, Mecasys, Daejeon, Republic of Korea).

The antifouling performance of the selected HF membranes was investigated using several processes. First, the membrane was filtered using DI water as the feed solution for 30 min to obtain the pure water flux (*J*_0_) of the virgin membrane. The feed solution was then exchanged with HA solution for 60 min, and the permeate flux was labeled *J_f_*. After filtration, the fouled membrane was cleaned by flushing with DI water for 20 min. Finally, the pure water flux (*J_c_*) of the cleaned membrane was measured using DI water for 30 min. The flux recovery ratio (FRR), total resistance (*R_t_*), intrinsic membrane resistance (*R_m_*), reversible resistance (*R_r_*), and irreversible resistance (*R_ir_*) were calculated using the following equations:

(5) $$\mathrm{FRR}=\left(\frac{J_{c}}{J_{0}}\right)\times 100\%,$$

(6) $$R_{t}=\frac{\Delta P}{\mu J_{f}}=R_{m}+R_{r}+R_{ir},$$

(7) $$R_{m}=\frac{\Delta P}{\mu J_{0}},$$

(8) $$R_{r}=R_{t}-\frac{\Delta P}{\mu J_{c}},$$

(9) $$R_{ir}=\frac{\Delta P}{\mu J_{c}}-R_{m},$$

where μ is the viscosity of permeate.

## 3. Results and Discussion

### 3.1. Characterization of CNT and FCNT

**Figure 2:**
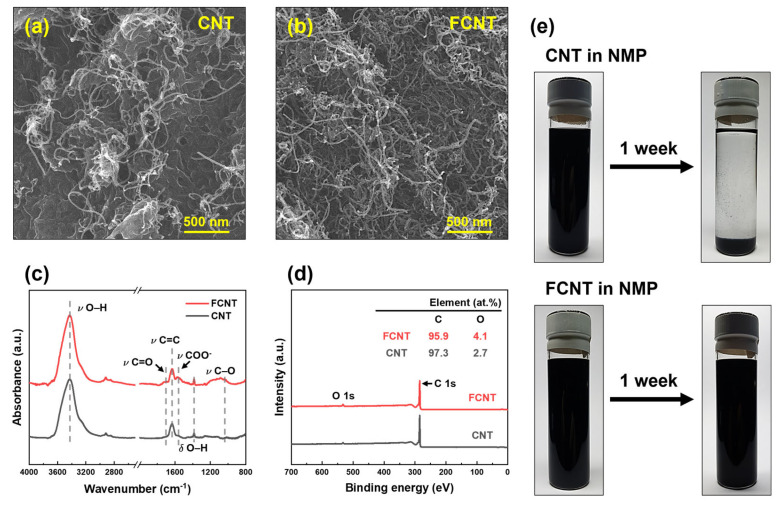
Characteristics of CNT and FCNT. (**a**,**b**) Microstructure. (**c**) ATR-FTIR spectra. (**d**) XPS spectra. (**e**) Dispersibility and stability.

The microstructure of the CNT and FCNT was characterized using FESEM (Figure 2a,b). Both CNT and FCNT have 10–15 nm diameter tubular structures with high aspect ratios. Compared to CNT, FCNT exhibited shorter tube lengths owing to fragmentation in the acid functionalization process [17,32]. This fragmentation causes open end tips, increasing the surface area and dispersibility of the FCNT in solvents [32,35]. In addition, after acid functionalization, bulk material, such as metal catalysts and impurities originating from the CNT production process, clearly disappeared from the FCNT. The ATR-FTIR spectra of the CNT and FCNT are shown in Figure 2c. After acid functionalization, carboxylic acid (–COOH) peaks appeared at 1040, 1570, and 1750 cm^−1^, corresponding to –CO, –COO^−^, and –C=O stretching vibrations, respectively. Moreover, the intensity of the -OH peak increased slightly in the ATR-FTIR spectrum of the FCNT. To investigate changes in the chemical composition after acid functionalization of the CNT, XPS analysis was carried out on CNT and FCNT (Figure 2d). Carbon and oxygen peaks are observed in both XPS spectra. The oxygen peak can be attributed to impurities and defects in CNT throughout the production and purification processes [36,37]. After acid functionalization, the oxygen content increased from 2.7 to 4.1% owing to the grafting of carboxylic acid groups onto the surface of the CNT. Both the ATR-FTIR and XPS results confirm that the CNT were successfully functionalized by acid treatment.

The dispersibility and stability of the CNT and FCNT were tested by dispersing them in NMP solvent. A certain amount of CNT and FCNT was added to NMP, and the CNT/NMP and FCNT/NMP mixtures were treated by ultrasonication for 90 min. Subsequently, the mixtures were allowed to stand for one week to compare the dispersibility and stability of the CNT and FCNT in NMP. As shown in Figure 2e, immediately after ultrasonication, both CNT and FCNT exhibited good dispersion in the NMP without any aggregation. After one week, the CNT settled with aggregation due to low stability in NMP. However, the FCNT still showed good dispersion in the NMP, without any settling or aggregation. This is due to increased polar–polar interaction between the carboxylic acid groups of the FCNT and the NMP, and the shortened tube lengths [35,38]. This enhancement of dispersibility and stability implies that the FCNT will show good dispersion and stability during the dope solution preparation and spinning processes.

### 3.2. Characterization of PES and FCNT/PES Nanocomposite HF Membranes

#### 3.2.1. Microstructure

**Figure 3:**
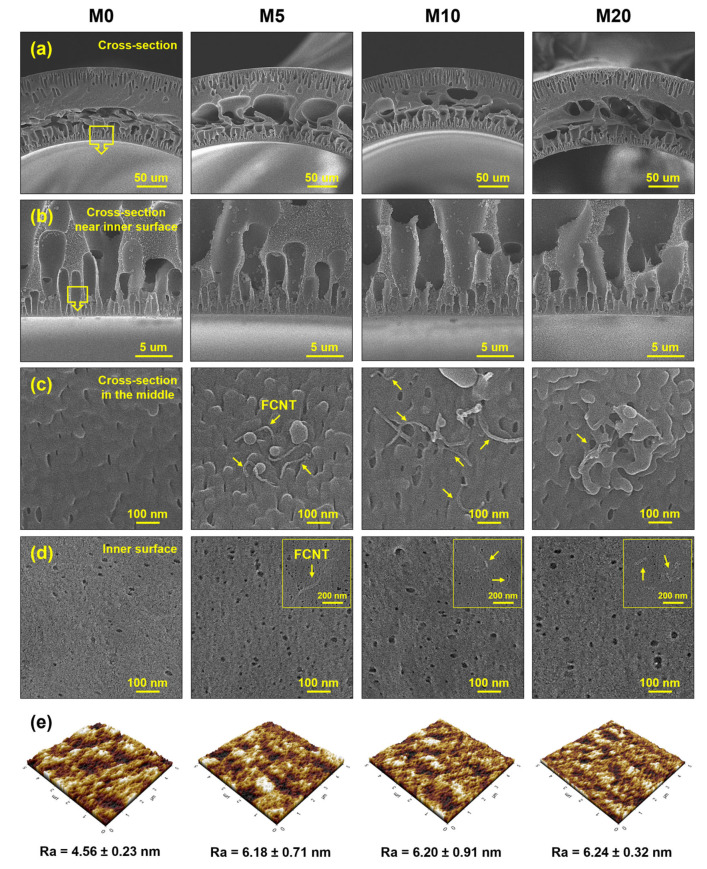
Microstructure of PES and FCNT/PES nanocomposite HF membranes. (**a**–**c**) Cross-section. (**d**) Inner surface. (**e**) Inner surface roughness.

The microstructure of the HF membranes is shown in Figure 3a–d. All the HF membranes have similar asymmetric cross-sectional microstructures. The membranes consist of finger-like macrovoids in the inner and outer layers, and large macrovoids and sponge-like structures in the middle section. Finger-like macrovoids in the inner and outer layers are formed because of the rapid instantaneous liquid–liquid demixing [35,39,40]. The finger-like macrovoids in the inner layer are connected to a large macrovoid in the middle section because the dope solution on the inner layer immediately contacts the bore fluid after spinning from the spinneret. In contrast, the dope solution on the outer layer passes through the air-gap region before entering the coagulation bath. Therefore, the different sizes and lengths of the finger-like macrovoids originate from different coagulation times. A sponge-like structure in the middle section was also formed owing to delayed demixing [39,40,41]. Compared to the pristine PES HF membrane (M0), the FCNT/PES nanocomposite HF membranes (M5, M10, and M20) exhibit larger finger-like macrovoids owing to rapid demixing during phase inversion [6,17,42]. The FCNT in the dope solution increase the hydrophilicity of the dope solution, resulting in an increase in its thermodynamic instability [17,39,40]. As shown in Figure 3c, all the FCNT/PES nanocomposite HF membranes contain FCNT inside the membrane; however, aggregated FCNT are observed in M20. The FCNT/PES nanocomposite HF membranes show a larger surface pore diameter and higher surface porosity than the pristine PES HF membrane (Figure 3d). As mentioned above, this result is due to blended FCNT inducing rapid demixing during phase inversion. A detailed analysis of the pore diameter and porosity of the fabricated HF membranes is presented in Section 3.2.3.

The roughness of the membrane surface affects filtration and antifouling performance. Therefore, the surface roughness of the fabricated HF membranes was measured by AFM. Average roughness (R_a_) was used as a quantitative parameter to compare the surface roughness of each membrane. The FCNT/PES nanocomposite HF membranes exhibited higher R_a_ values than the pristine PES HF membrane because of increased pore diameter and porosity of the membrane surface [11,43]. The increased roughness may contribute to a higher flux, owing to the increased surface area [11,44]. However, a higher membrane surface roughness might increase the fouling potential on the membrane surface because of the foulants accumulating in the valleys [45].

#### 3.2.2. Chemical Properties

**Figure 4:**
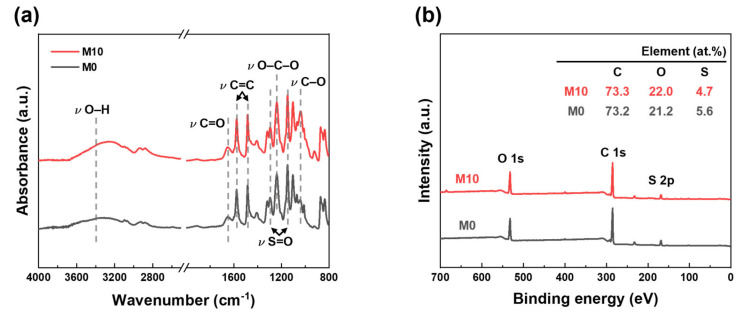
Chemical properties of PES and FCNT/PES nanocomposite HF membranes. (**a**) ATR-FTIR spectra. (**b**) XPS spectra.

M0 (pristine PES HF) and M10 (FCNT/PES nanocomposite HF) membranes were selected to investigate the effect of FCNT blending on the chemical properties of the membrane. The ATR-FTIR spectra of M0 and M10 are shown in Figure 4a. The characteristic peaks of PES are observed at 1149, 1240, 1294, 1484, and 1577 cm^−1^, corresponding to symmetric S=O, C–O–C, asymmetric S=O stretching vibrations, and aromatic C=C stretching, respectively. Additionally, –C=O and –OH peaks are observed in both ATR-FTIR spectra due to residual PVP in the membrane [46,47]. The –C=O and –OH peak intensities of M10 increase because of the blended FCNT. The surface chemical composition of the M0 and M10 membranes was determined using XPS (Figure 4b). Compared to M0, M10 exhibits a higher oxygen content owing to the blended FCNT in the membrane. However, the sulfur content decreased. Through ATR-FTIR and XPS analyses of M0 and M10, we can confirm that FCNT were successfully blended in the HF membrane.

#### 3.2.3. Physical Properties

**Figure 5:**
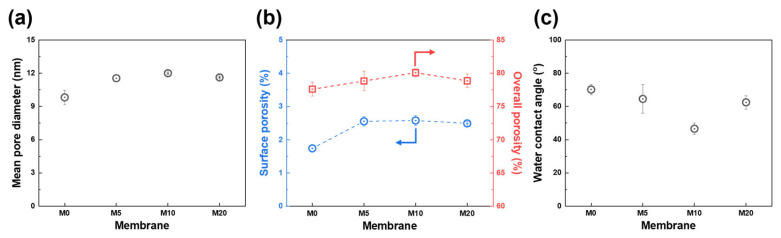
Physical properties of PES and FCNT/PES nanocomposite HF membranes. (**a**) Mean pore diameter. (**b**) Surface (blue) and overall (red) porosity. (**c**) Water contact angle.

The pore properties of the fabricated PES HF membranes are shown in Figure 5a,b. The surface mean pore diameter increased from 9.8 to 12.0 nm as the FCNT concentration increased from 0 to 1 wt%. This is because as the FCNT concentration increases, the demixing rate increases during phase inversion, resulting in an increase in pore size [6,11,40]. However, with a further increase in the FCNT from 1 to 2 wt%, aggregation of FCNT in the membrane becomes the main reason for decreasing pore size [11,17,42].

The effect of FCNT blending on the hydrophilicity of the membrane surface was investigated. Hydrophilicity was determined by water contact angle measurements. Typically, a lower water contact angle implies higher hydrophilicity, and a more hydrophilic membrane surface exhibits better filtration and antifouling performance [1,44,48]. As shown in Figure 5c, the water contact angle decreases from 70.2° to 46.6° as the blended FCNT concentration increases from 0 to 1 wt%. However, when the blended FCNT concentration increases over 1 wt%, the water contact angle increases from 46.6° to 62.5° owing to aggregation of the FCNT, which is consistent with the microstructure analysis results in Section 3.2.1. The water contact angle measurements confirm that the blended FCNT enhance the hydrophilicity of the membrane surface. An explanation for this result is that the blended FCNT spontaneously migrate to the membrane surface during the phase inversion process, resulting in hydrophilic carboxyl groups on the FCNT surface, which increase the hydrophilicity of the membrane surface [1,17,35,38].

### 3.3. Filtration and Antifouling Performance of PES and FCNT/PES Nanocomposite HF Membranes

#### 3.3.1. Filtration Performance

**Figure 6:**
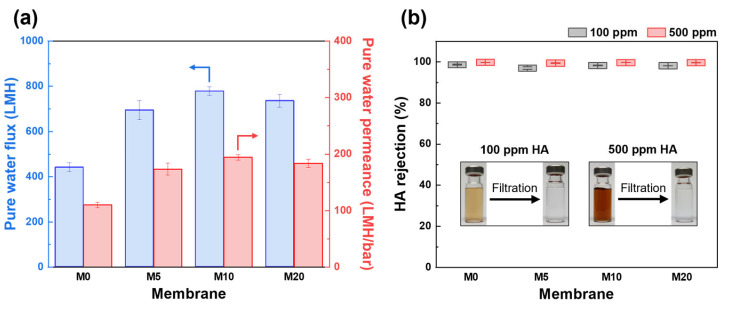
Filtration performance of PES and FCNT/PES nanocomposite HF membranes. (**a**) Pure water flux (blue) and permeance (red). (**b**) HA rejection.

As shown in Figure 6a, the pure water flux and permeance of the FCNT/PES nanocomposite HF membrane were significantly higher than those of the pristine PES HF membrane (M0). The pure water flux and permeance of M0 were 440.4 LMH and 110.1 LMH/bar, respectively, while those for the M10 were 777.3 LHM and 194.3 LMH/bar, respectively. This enhancement is attributed to the increased pore size and porosity, as well as the enhanced hydrophilicity of the membrane [6,17,42].

The rejection performance of the fabricated HF membranes was evaluated using HA solutions (100 and 500 ppm) as feed solutions. As shown in Figure 6b, the rejection performance using 100 ppm HA solution as a feed solution was 98.6, 97.0, 98.3, and 98.1% for the M0, M5, M10, and M20, respectively. Using a 500 ppm HA solution as the feed solution, all membranes exhibited a slightly higher rejection value of over 99.0%. When a higher concentration of HA solution is used as a feed, more HA molecules are deposited on the membrane surface, resulting in increased steric hindrance and increased repulsion forces by increasing the negative charge on the membrane surface [49,50]. After filtration using M10, the HA solution became clear and transparent (insert of Figure 6b). The FCNT/PES nanocomposite HF membranes did not sacrifice any rejection performance, even though the water permeance was higher than that of the pristine PES HF membrane. This is due to the improved hydrophilicity and negative charge of the membrane surface containing FCNT, resulting in HA being more effectively repelled from the membrane surface [6,45,48].

#### 3.3.2. Antifouling Performance

**Figure 7:**
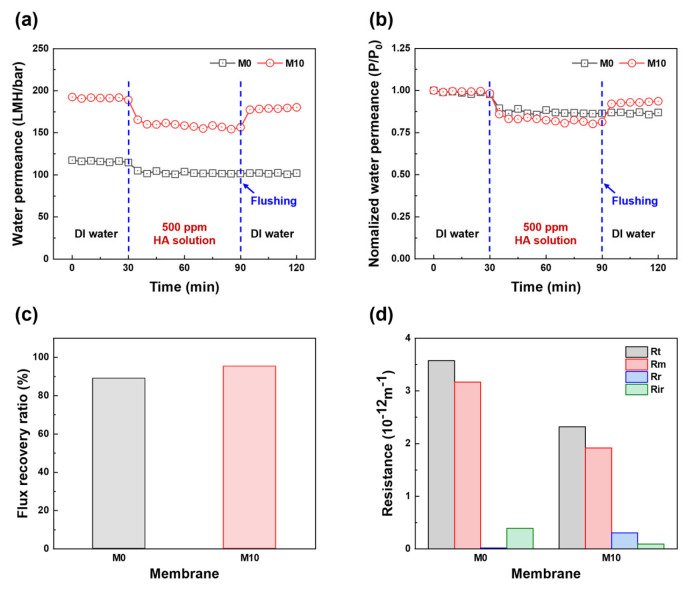
Antifouling properties of PES and FCNT/PES nanocomposite HF membranes. (**a**) Water permeance as a function of time. (**b**) Normalized water permeance as a function of time (P/P_0_: water permeance relative to the initial water permeance). (**c**) Flux recovery ratio. (**d**) Resistance profiles.

Based on the filtration performance of the fabricated HF membranes, M10 was selected to investigate the antifouling performance and M0 was used as the control membrane. As shown in Figure 7a, the selected membranes were tested using DI water and a 500 ppm HA solution. After replacing the HA solution as a feed solution, both M0 and M10 exhibited an immediate water permeance decline from 114.7 to 105.0 LMH/bar and from 189.0 to 165.5 LMH/bar, respectively, due to the deposition and adsorption of HA on the membrane surface. Subsequently, the water permeance of both membranes continuously decreased to 101.5 and 156.5 LMH/bar for M0 and M10, respectively, during HA solution filtration. Although the water permeance decreased, the permeance of M10 was still higher than that of M0, owing to its large pore size, high porosity, and hydrophilicity. After cleaning, the water permeance value of M10 was close to the initial value, whereas the water permeance of M0 hardly recovered. In addition, the normalized water permeance, FRR, and resistance profiles (i.e., Rt, Rm, Rr, and Rir) were calculated from the HA filtration test results to investigate the fouling behavior and antifouling properties. As shown in Figure 7b, the normalized water permeance clearly shows a change in permeance behavior compared to the initial water permeance of each membrane during the fouling test. After replacing the HA solution, M10 exhibited a higher normalized water permeance reduction than M0. This is because the larger pore size, higher porosity, and rougher surface of the membrane indicate a higher fouling tendency [1,51]. However, the FRR of M10 was higher than that of M0. As shown in Figure 7c, the FRR values are 89.0 and 95.4% for M0 and M10, respectively. Figure 7d shows the resistance profiles of the membrane and fouling layer. M0 has a higher Rm than M10 because of its smaller pore size, lower porosity, and hydrophilicity. The resistance of the fouling layer on M0 mainly consists of Rir, whereas the resistance of the fouling layer on M10 mainly consists of Rr. The hydrophilic membrane surface forms a hydration layer on the membrane surface owing to the blended FCNT, which diminishes the adhesion force between the HA and membrane surface, resulting in the fouling layer being washed easily from the membrane surface by the cleaning process [6,11,42,52,53].

**Figure 8:**
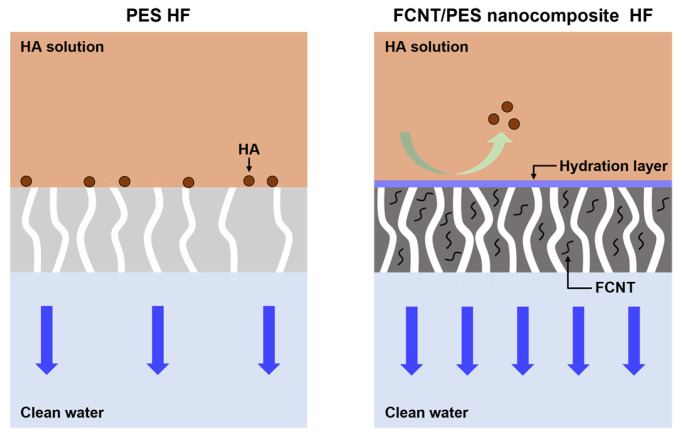
Schematic illustration of filtration and antifouling mechanisms of PES and FCNT/PES nanocomposite HF membranes.

The FCNT/PES nanocomposite HF membrane exhibited a higher filtration and antifouling performance than the pristine PES HF membrane. As illustrated in Figure 8, the blended FCNT increase surface pore size and porosity and increase the hydrophilicity of the membrane, resulting in the formation of a thin hydration layer on the membrane surface. Additionally, the FCNT on the membrane surface increase the negative charge density on the membrane surface. The resulting water permeance and antifouling properties are thus improved without sacrificing rejection performance.

## 4. Conclusions

In this study, we developed a high filtration and antifouling PES HF membrane for the UF process by blending FCNT into the membrane. The FCNT were prepared by acid functionalization of pristine CNT to increase their hydrophilicity, dispersibility, and stability. An FCNT/PES nanocomposite HF membrane was fabricated via a dry-jet wet spinning process. The FCNT/PES nanocomposite HF membrane exhibited a larger surface pore size, and increased surface porosity and hydrophilicity than the pristine PES HF membrane. As a result, the water performance increased from 110.1 to 194.3 LMH/bar without sacrificing any rejection performance. Additionally, the FCNT/PES nanocomposite HF membrane exhibited an excellent FRR value of 95.4%, which increased from 89.0% in the pristine PES HF membrane. The results of this study validated that FCNT blending in a PES HF membrane can improve the filtration and antifouling performance of the membrane. This study provides a feasible strategy for the development of high performance and antifouling PES HF membranes for the UF process.

## Figures and Tables

**Table 1:** Composition of dope solutions for FCNT/PES nanocomposite HF membranes.

Membrane	PES (wt%)	NMP (wt%)	PVP ^1^ (wt%)	FCNT ^2^ (wt%)
M0	17	83	1	-
M5	17	83	1	0.5
M10	17	83	1	1.0
M20	17	83	1	2.0

^1^ Mass ratio of PVP to total dope solution. ^2^ Mass ratio of FCNT to PES.

**Table 2:** Spinning parameters for FCNT/PES nanocomposite HF membranes.

Spinning Parameters	
Dope solution pressure (bar)	0.2
Bore fluid	DI water
Bore fluid flow rate (mL/min)	2.0
Air-gap length (cm)	5
Take-up speed (m/min)	free fall
External coagulation	Tap water
Spinneret dimensions (mm)	1.1–0.6
Spinning temperature (°C)	20–25
Humidity (%)	40–60

## Data Availability

Not applicable.
